# Influences of Sodium Lignosulfonate and High-Volume Fly Ash on Setting Time and Hardened State Properties of Engineered Cementitious Composites

**DOI:** 10.3390/ma14174779

**Published:** 2021-08-24

**Authors:** Anggun Tri Atmajayanti, Chung-Chan Hung, Terry Y. P. Yuen, Run-Chan Shih

**Affiliations:** 1 Department of Civil Engineering, National Cheng Kung University, 1 University Road, Tainan City 701, Taiwan; anggun.tri.atmajayanti@gmail.com; 2 Department of Civil Engineering, National Yang Ming Chiao Tung University, Hsinchu 300, Taiwan; terryyyp@nctu.edu.tw; 3 Department of Civil Engineering, National Central University, Taoyuan City 320, Taiwan; fofo_1018@hotmail.com

**Keywords:** ECC, fly ash, setting time, autogenous shrinkage, drying shrinkage, sodium lignosulfonate

## Abstract

Engineered Cementitious Composites (ECC) exhibit high ductility accompanied by multiple narrow cracking behavior under uniaxial tension. The study experimentally investigated the influence of sodium lignosulfonate and high volumes of fly ash (HVFA) on the properties of fresh and hardened ECC, with the experimental variables including the amounts of fly ash, polyvinyl alcohol (PVA) fibers, and sodium lignosulfonate. The test results were discussed extensively in terms of the initial and final setting times, compressive and tensile behavior, and drying and autogenous shrinkage. The results indicated that the initial and final setting times of ECC were increased along with the sodium lignosulfonate content of up to 1%**.** The drying shrinkage development was governed by the first 14 days. In addition, the major autogenous shrinkage developed for more than 28 days. The amounts of fly ash, PVA fibers, and sodium lignosulfonate considerably impacted the autogenous shrinkage. Moreover, it was found that the dosage of sodium lignosulfonate at 0.5% of the weight of Portland cement optimally reduced the shrinkage and enhanced the tensile strain capacity for ECC.

## 1. Introduction

High-performance fiber-reinforced cementitious composites (HPFRCCs) are characterized by pseudo-strain-hardening behavior accompanied by multiple narrow cracks when under uniaxial tension. They have attracted the increasing attention of researchers and engineers to a wide range of innovative applications in structural engineering due to their advantageous mechanical properties and durability [1,2,3,4,5,6,7,8,9,10,11]. Engineered Cementitious Composites (ECC), a unique class of HPFRCCs, feature a large strain capacity with self-controlled crack width [12,13,14,15,16,17,18,19,20,21,22,23]. Its tensile strain capacity can reach more than 3% [17,18,19,20,21,22,23,24,25,26], and its average crack width was reported to be less than 60 μm when the tensile strain demand was less than 1% [14]. The high strain capacity and multiple hairline cracking behavior of ECC are achieved by tailoring the fracture toughness of the matrix by excluding coarse aggregate and limiting the fine sand content [27,28]. Compared to traditional concrete materials, ECC usually contains a considerably higher cement content and lower aggregate content, which could aggravate drying and autogenous shrinkage. In particular, it was showed that the drying shrinkage strain of ECC can reach as high as $1200\times 10^{-6}$ to $1800\times 10^{-6}$ [29].

The shrinkage behavior is an important factor affecting the durability of cement-based materials [2,7,15,30,31,32,33,34,35,36,37,38,39]. Xinqi et al. [28] experimentally showed that the shrinkage of ECC at 14 days of age reached 90% of the shrinkage at 28 days of age. They concluded that the crack resistance of ECC was significantly affected by the shrinkage behavior within the age of 14 days. Sahmaran and Li. [40] used saturated lightweight fine aggregate in ECC as an internal curing agent. The autogenous shrinkage of ECC was reduced by 67% through the replacement of 20% silica sand with saturated lightweight fine aggregate. Zhang et al. [41] showed that the replacement of Type I Portland cement with low-shrinkage cement in ECC effectively reduced the shrinkage, with the 28-day drying shrinkage strain of only $109\times 10^{-6}$ to $242\times 10^{-6}$. Nath and Sarker [27] indicated that partially replacing Portland cement with FA did not warrant a lower drying shrinkage unless the free moisture content in the matrix was reduced. Wang and Li [42] used FA and bottom ash in ECC to improve the sustainability and strain-hardening behavior of ECC. The results demonstrated a robust tensile strain capacity of 3–4% and tensile strength above 4.5 MPa while material sustainability indexes were significantly improved.

Due to the relatively low water-binder ratio of ECCs and the absence of coarse aggregate, high-range water reducer (HRWR) or superplasticizer is often added to the mixture to achieve the desired workability. Among different types of water-reducing agents, lignosulfonates (LSs), which can be a by-product derived from the sulfite wood-pulping process [43], has been widely used due to its relatively low price [44] and effectiveness in reducing the required water content for cement-based composites [45]. While the inclusion of lignosulfonate (LS) generally led to a higher compressive strength for concrete due to the lower amount of water needed [46,47,48,49], Topcu and Atesin [50] showed that the dosage of LS of greater than 1% reduced the compressive strength of concrete.

In addition to reducing the required water, LS is also known as an effective retarder for fresh concrete [47,51,52]. The mechanisms of OPC hydration retardation are due to the interaction of the Ca^2+^ ions with the negative charged anions of LS and the inhibited conversion of needle-shaped AFt (ettringite) to AFm (monosulfate) under the presence of LS [53]. While the reduced availability of Ca^2+^ decreases the formation of crystalized calcium hydroxide (CH), a hydrophobic film of the LS anion adsorbed onto the cement particle surface would restrict the water access to the particles. On the other hand, the LS anions absorbed onto AFt would also inhibit its further crystallization to AFm. Vikan [54] concluded that LS was more effective than polyacrylate and naphthalene sulfonate formaldehyde in prolonging the setting time. Topcu and Atesin [50] demonstrated that the addition of LS at 5% extended the initial and final setting times for 2 h and 6 h, respectively. Cabrera et al. [55] showed that the addition of LS led to a lower plastic shrinkage due to the reduced surface tension.

Despite the popularity of LS in ordinary cement concrete, there is still a knowledge gap in the effect of LS on the properties of fresh and hardened ECC. In addition, the studies on the autogenous shrinkage behavior of ECC with high volumes of FA (HVFA-ECC) were scarce in the literature. Therefore, a systematic experimental study was conducted to investigate the influences of fly ash, PVA fibers, and lignosulfonate in the properties of fresh and hardened ECC. The results were discussed in terms of the initial and final setting times, compressive and tensile behavior, and drying and autogenous shrinkage.

## 2. Experimental Program

### 2.1. ECC Mixtures and Preparation

Eight ECC mixtures were developed to assess the influence of varying amounts of FA, short fibers, and sodium lignosulfonate (NLS) in the properties of ECC. The water-to-binder (w/b) ratio was 0.35 for all ECC mixtures. The mixture proportions are listed in Table 1. The components included Type I ordinary Portland cement (OPC), Class F fly ash (FA), silica sand (SS) with particle sizes ranging from 0.105 mm to 0.210 mm, polycarboxylate superplasticizer (PCE), water, NLS, and polyvinyl alcohol (PVA) fibers. The length and diameter of the PVA fibers were 12 mm and 39 μm, respectively. The fibers had a tensile strength of 1600 MPa, a density of 1300$\;\mathrm{kg}/m^{3}$, an elastic modulus of 41 GPa, and a maximum elongation ratio of 6%. The adopted NLS mainly consisted of silicon dioxide, calcium oxide, sulfate, and aluminum oxide.

The mixing procedure followed the steps suggested by Hung et al. [25] that evaluated the medium-term self-healing of ECC with varying amounts of fly ash and exposure durations. All dry particles were pre-mixed in a Hobart mixer. Then, the chemical additives and half of the required water were mixed and soon added to the mixer [56]. After that, the PVA fibers and the remaining water were added to the mixer. Table 2 shows the detailed compositions of adopted NLS and FA. The name of an ECC mixture in Table 1 consists of three parts: the FA-to-OPC (FA/C) ratio (by weight), volume fraction (*V_f_*) of PVA fibers, and NLS-to-OPC ratio (by weight). For example, FA1.2-2%-0.5% represents the ECC mixture with the FA/C ratio of 1.2, *V_f_* = 2%, and the NLS-to-OPC ratio of 0.5%.

### 2.2. Evaluation Methods

#### 2.2.1. Initial and Final Setting Time

**Figure 1:**
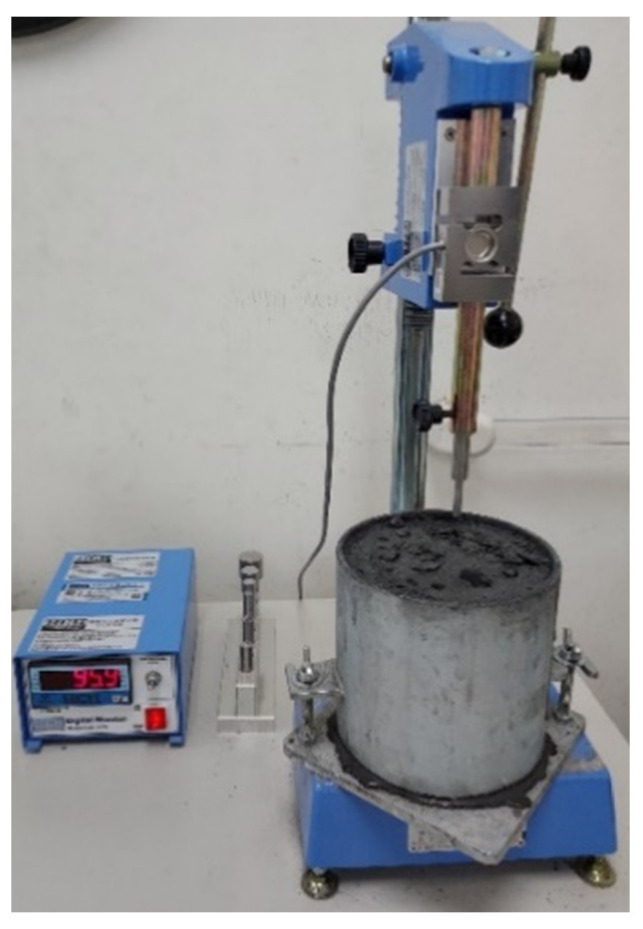
Evaluation of setting time by penetration resistance.

The setting time of the fresh ECC was obtained using penetration tests in accordance with ASTM C403M-08 Standard Test Method for Time of Setting of Concrete Mixtures by Penetration Resistance [57], as shown in Figure 1.

The ambient temperature was controlled to be 20 ± 2 °C throughout the tests. Initial and final setting times were determined as the duration required for the penetration resistance to reach 3.5 and 27.6 MPa, respectively.

#### 2.2.2. Drying and Autogenous Shrinkage

**Figure 2:**
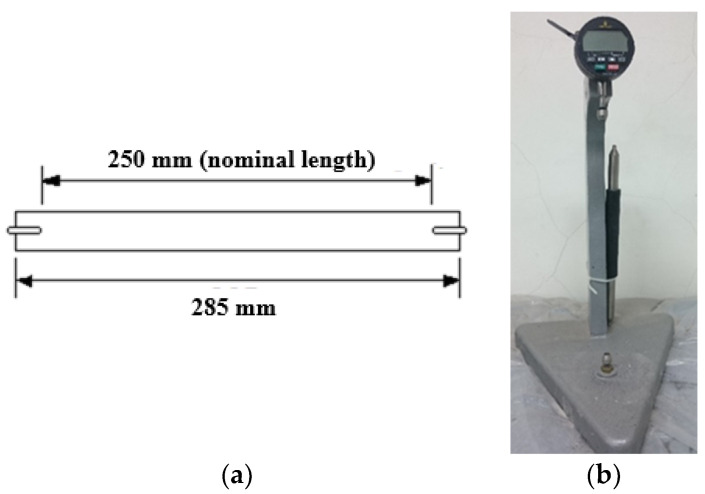
Evaluation of shrinkage: (**a**) Shrinkage specimen; (**b**) Digital length comparator.

The drying shrinkage of ECC was examined using four prismatic samples with dimensions of 25 × 25 × 285 mm^3^ according to ASTM C490M-17 Standard Practice for Use of Apparatus for Determination of Length Change of Hardened Cement Paste Mortar and Concrete [58]. Before the material samples were cast, two steel gauge studs were inserted at both ends of the mold, which led to a gauge length of 250 mm (comply with ASTM C490M-17), as shown in Figure 2a. The samples were demolded 24 h after casting and then cured for 3 days in water at 20 ± 2 °C. The length of the samples after 3 days of water curing was taken as the initial length. After that, the samples were placed in a constant humidity incubator with a controlled 50% RH and 20 ± 2 °C. Then, the length of the samples was measured daily in the first 7 days and then every week until the 90th day. For determination of the autogenous shrinkage, another four prismatic samples were prepared and examined in a similar procedure as for the drying shrinkage, except the molds were sealed with aluminum foils as soon as the casting was done to prevent the loss of moisture within the samples due to evaporation.

A digital length comparator, as shown in Figure 2b, with a measurement range of 15.2 mm and a resolution of 2.54 μm was adopted to measure the length of the samples. The measurement was made by sliding the feet of the comparator to contact the top of each gauge stud. The shrinkage strain of the samples was calculated as *ε_s_* = $\left(L_{0}-L_{t}\right)$/*G*, where $L_{0}$ and $L_{t}$ are the lengths measured at the initial condition and the specified time, and *G* is the gauge length.

#### 2.2.3. Compressive and Tensile Properties

**Figure 3:**
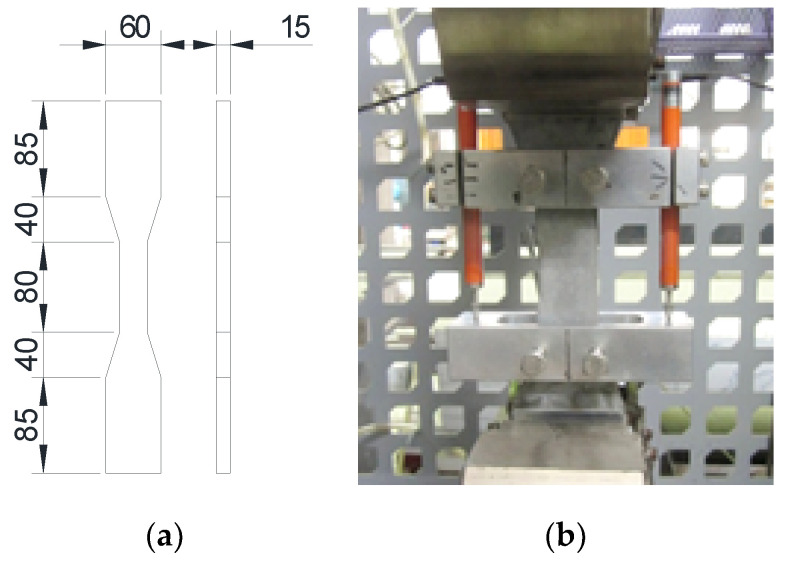
Tensile test on the dog-bone-shaped specimen: (**a**) Specimen dimensions; (**b**) Test setup.

The compressive strength of the ECC materials was examined using standard cylindrical specimens with a diameter of 100 mm and a height of 200 mm and a procedure consistent with ASTM C39 Standard Test Method for Compressive Strength of Cylindrical Concrete Specimens [59]. The tensile properties of the ECC were determined using uniaxial tensile tests on dog-bone-shaped specimens based on the Japan Society of Civil Engineers (JSCE) Recommendations for Design and Construction of High Performance Fiber Reinforced Cement Composites with Multiple Fine Cracks (HPFRCC) [60] with geometrical dimensions, shown in Figure 3. Before the tensile tests, thin aluminum plates were glued to both ends of the dog-bone-shaped specimens for the loading system to effectively grip the specimen. Two linear variable displacement transducers (LVDTs) were placed on both sides of the specimen to measure the tensile deformation of an 80 mm long-gauge region. Both compressive and tensile tests were carried out using a displacement control procedure with a consistent loading rate of 0.5 mm/min. For the preparation of the specimens, they were demolded 24 h after casting. Then, the specimens were cured in water until one day prior to the tests, which was followed by air curing at a controlled temperature of 20 ± 2 °C.

## 3. Experimental Results and Discussions

### 3.1. Setting Time

**Figure 4:**
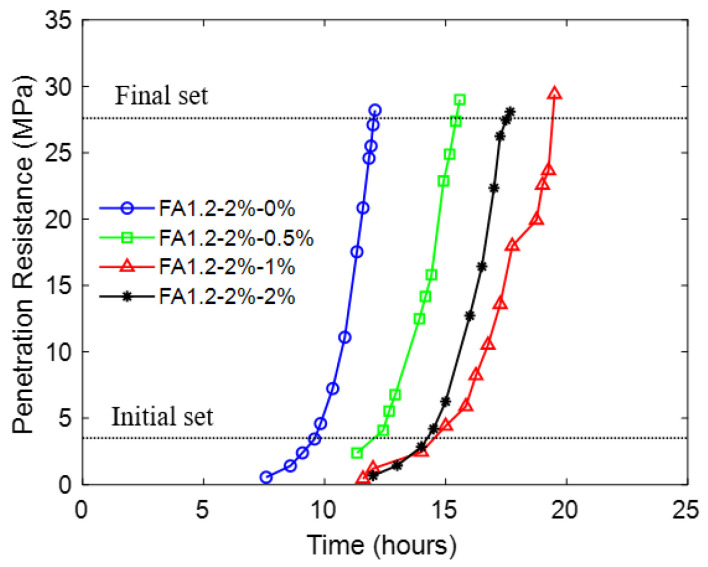
Influence of NLS on the penetration resistance of ECC.

Figure 4 shows the relationship between the penetration resistance and time for the series of FA1.2-2% mixtures with different amounts of NLS.

The results show that increasing the NLS dosage from 0% to 1.0% increased the initial setting time from 9.6 hrs to 15.5 hrs and the final setting time from 12.1 h to 19.7 h. However, a further increase in NLS from 1.0% to 2% had negligible influence in the initial setting time and reduced the final setting time. The duration between the initial and final sets also had a similar trend with the NLS content. This result implies that when the NLS content was increased from 0% to 1.0%, more flocculated cement grains were disassembled, and hence more entrapped water was released. When the NLS content was more than 1%, flocculated cement grains were nearly fully disassembled, and the initial setting time remained essentially unchanged with the addition of NLS. The reduction of the final setting time of the mixture with 2.0% NLS would be explained by the increase in particle surface area with increasing NLS dosage [56]. Hence, when the hydration products began to develop after the initial set, the larger surface area could provide more nucleation sites for the crystallization.

### 3.2. Drying Shrinkage

**Figure 5:**
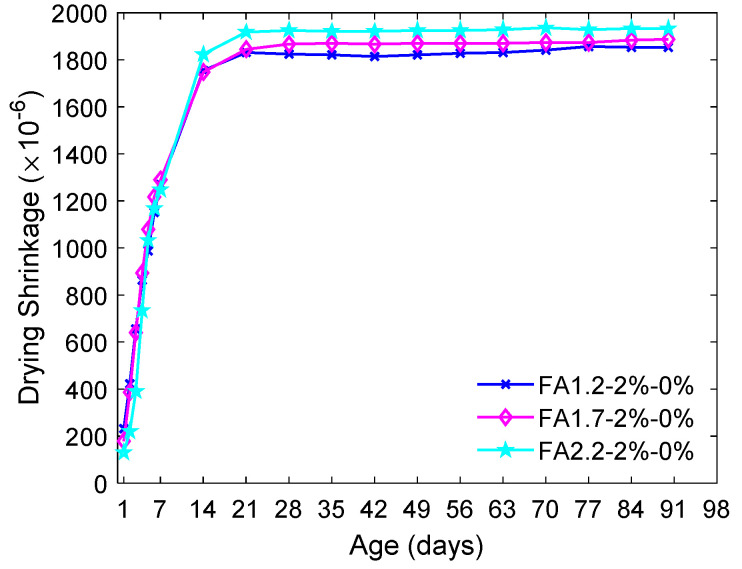
Influence of FA content in the drying shrinkage of ECC.

Figure 5 shows the drying shrinkage of the ECC with *V_f_* = 2% and different FA/C ratios. 

In the early stage, the drying shrinkage increased steadily and rapidly to about 1200 μ at 7 days. After that, although the shrinkage growth rate slightly reduced, the drying shrinkage continued to increase to about 1800 μ at 14 days, which was approximately 90% of the 90-day drying shrinkage. Then, the drying shrinkage gradually stabilized and essentially became constant after 20 days. Overall, increasing the FA/C ratio from 1.2 to 2.2 had a negligible influence on the drying shrinkage before the 14th day. At 90 days, it slightly increased the total drying shrinkage from 1852 μ to 1932 μ. The increasing drying shrinkage due to the higher FA/C ratio could be attributed to multiple factors. Firstly, the capillary stress in the ECC with a higher FA/C ratio could be higher due to the refined microscopic pore structure [29,61,62], which increased the loss of water from capillary voids [62]. Moreover, a higher FA/C ratio tended to reduce the elastic modulus of the solid skeleton and the bond between PVA fibers and the surrounding matrix [42], thus leading to a higher drying shrinkage [63].

**Figure 6:**
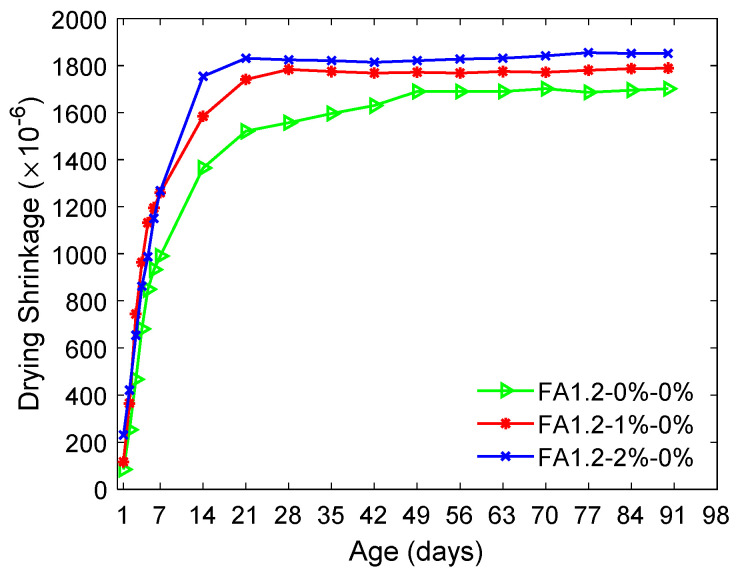
Influence of fiber content in the drying shrinkage of ECC.

The influence of *V_f_* in the drying shrinkage of ECC is illustrated in Figure 6. 

It can be seen that the ECC with a greater amount of PVA fibers appeared to have a higher shrinkage rate prior to the 14th day. At the age of 90 days, increasing *V_f_* from 0% to 2% slightly increased the drying shrinkage of ECC from 1702 μ to 1852 μ, about a 10% magnification. This could be due to the increased entrained air [64] following the increase in fiber content and the entrained air would lead to larger shrinkage [65].

**Figure 7:**
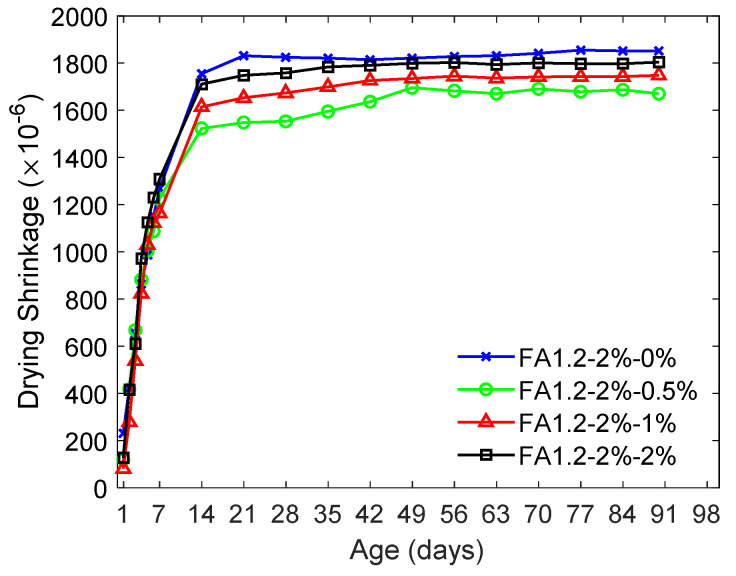
Influence of NLS content in the drying shrinkage of ECC.

Figure 7 shows the influence of NLS on the drying shrinkage of ECC. The inclusion of an NLS dosage of 0.5% led to an approximate 10% reduction in the drying shrinkage. Bishop and Barron [66] experimentally showed that the presence of LS accelerated ettringite formation due to the hydration of C_3_A. Moreover, LS involves the formation of a semipermeable layer on cement grains, which could slow down the diffusion of pore solution [66] and alleviate volume reduction due to drying shrinkage. Because of these possible mechanisms, the drying shrinkage was reduced after the inclusion of NLS at 0.5%. Nevertheless, the beneficial effect in reducing the drying shrinkage gradually decreased with the increased NLS content from 0.5% to 2%. For the ECC with 2% NLS, its drying shrinkage was similar to the result of the ECC without NLS. It is noted that the “consumption” of LS by cement grains forming the semipermeable layer is the largest at a specific LS dosage [43,56]. After that dosage, the uptake of LS by the cement grains as well as the semipermeable layer will decrease. Hence, among the considered mixtures, 0.5% NLS would apparently lead to the maximum LS uptake by cement. Moreover, high dosage LS can also lead to increased air entrainment [56] that can similarly result in high shrinkage [65].

### 3.3. Autogenous Shrinkage of ECC

**Figure 8:**
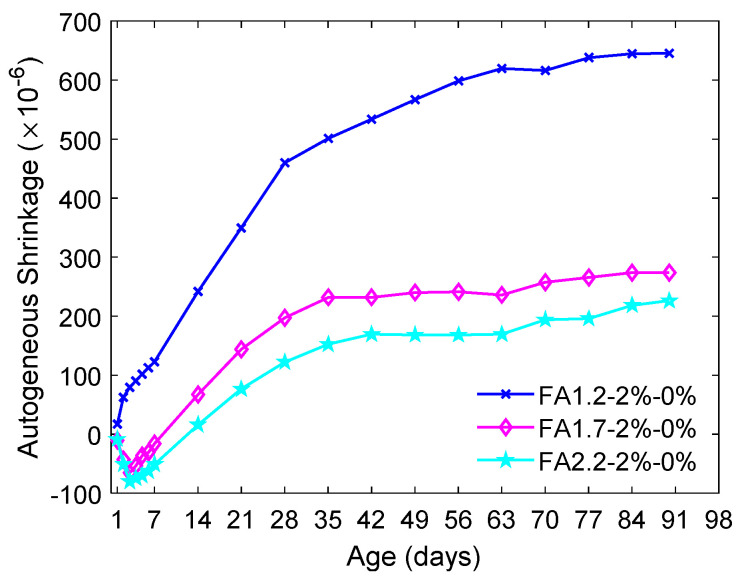
Autogenous shrinkage of ECC with different FA contents.

Figure 8 shows the impact of the FA/C ratio on the autogenous shrinkage of ECC at different ages. It can be seen that the autogenous shrinkage of ECC rapidly developed within 28 days. Then, the autogenous shrinkage of ECC became relatively stable, especially for the ECC with a higher FA/C ratio. The comparison results of Figure 5 and Figure 8 indicated that ECC had a substantially higher drying shrinkage than the autogenous shrinkage at the age of 90 days. While increasing the FA/C ratio from 1.2 to 2.2 only slightly increased the drying shrinkage (Figure 5), it considerably reduced the 90-day autogenous shrinkage from about 640 µ to 220 µ (Figure 8), approximately a 65% reduction.

Overall, a high FA/C ratio led to an effective reduction in the autogenous shrinkage of ECC due to the reduced contribution from cement hydration caused by a lowered cement content. It is also widely known that using FA to partially replace cement is able to reduce the water demand in concrete due to the ball bearing effect of round FA particles. As a result, more free water retained in the ECC when the FA/C ratio was higher, which helped cover the surfaces of unhydrated particles and reduce self-desiccation. Moreover, autogenous shrinkage is the result of the change in the macro volume of cement paste, which is dependent on the volumetric percentage of pores. A higher FA/C ratio reduced the volumetric percentage of the pores due to enhanced pozzolanic reaction [67,68], thus reducing the autogenous shrinkage of ECC.

**Figure 9:**
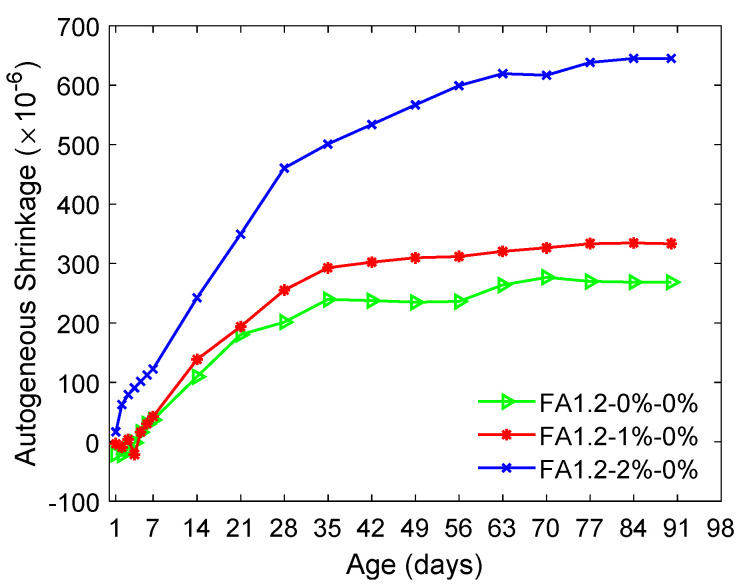
Autogenous shrinkage of ECC with different fiber contents.

Figure 9 presents the effect of fiber contents on the autogenous shrinkage of ECC with FA/C = 1.2. The autogenous shrinkage increased along with the fiber content. Notably, while the 90-day autogenous shrinkage was increased by about 20% when *V_f_* was increased from 0% to 1%, it was substantially magnified by approximately 100% when *V_f_* was further increased to 2%. This can be attributed to the presence of hydrophilic PVA fibers that absorbed some free water from cement and hydration products [69], thus aggravating the self-desiccation. In addition, air voids and porosity in fiber-reinforced concrete increased rapidly along with the fiber content [70], which could intensify the change in the capillary pressure induced by self-desiccation in the cement matrix.

**Figure 10:**
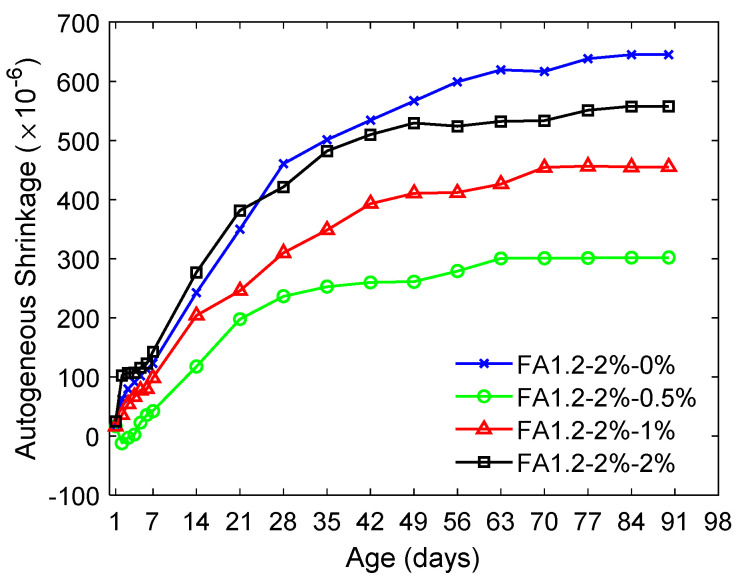
Autogenous shrinkage of ECC with different amounts of NLS.

Figure 10 shows that the inclusion of NLS reduced the autogenous shrinkage of the FA1.2-2% mixtures. This was due to the inclusion of NLS-retarded cement hydration [71], which reduced the hydration heat and the autogenous shrinkage [72]. The 90-day autogenous shrinkage of the FA1.2-2% mixtures ranged between 300 µ and 650 µ when the dosage of NLS was 0-2%. The inclusion of 0.5% NLS led to the least autogenous shrinkage, which could be attributed to the slowed cement hydration due to the semipermeable layer on cement grains due to the LS uptake as explained above. It greatly reduced the 90-day autogenous shrinkage to around 300 µ, about a 50% reduction compared to the case when there was no NLS.

### 3.4. Mechanical Properties of the ECC Materials

#### 3.4.1. Compressive Strength

**Figure 11:**
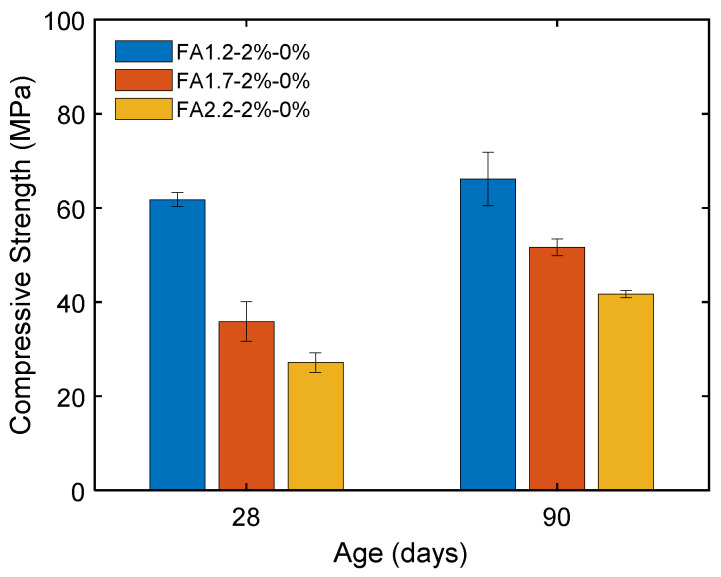
Compressive strengths of ECC with different amounts of FA.

Figure 11 shows that a higher FA/C ratio led to a lower compressive strength of ECC at both 28 and 90 days due to the reduced contribution from cement hydration caused by a lowered cement content. Increasing the FA/C ratio from 1.2 to 2.2 caused the 28-day compressive strength to substantially decrease from 62 MPa to 27 MPa. Nevertheless, the ECC with a higher FA/C ratio had a more substantial strength development at the later age due to the pozzolanic reaction. As a result, the reduction in the compressive strength due to the use of a higher FA/C ratio became less significant when the age was increased to 90 days.

**Figure 12:**
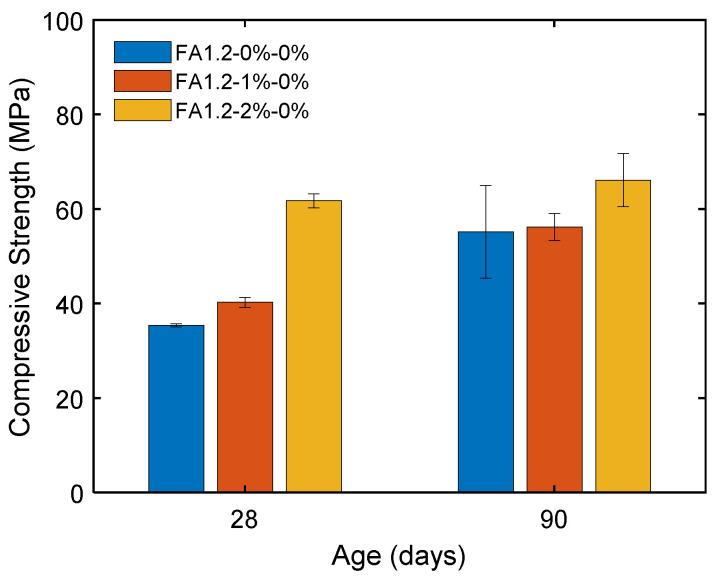
Compressive strengths of ECC with different amounts of fibers.

Figure 12 shows the effect of fiber content on the compressive strength of ECC. It can be seen that the compressive strength was generally higher with a greater *V_f_*. Compared to the case with *V_f_* = 1%, the enhancement in the compressive strength was more significant when *V_f_* = 2%. The 28-day strength was considerably enhanced from 35 MPa to 66 MPa when *V_f_* was increased from 0% to 2%. This could be attributed to the bridging of the propagating wing-cracks in the cementitious matrix by fibers under compression.

**Figure 13:**
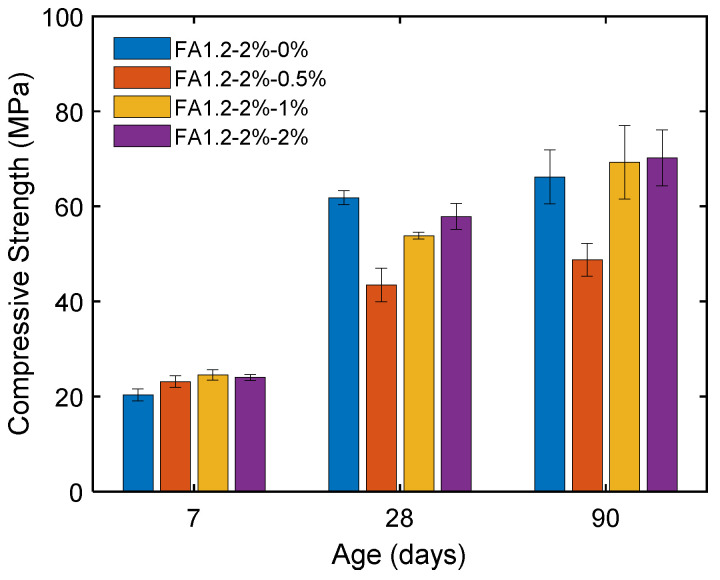
Influence of NLS on the compressive strength of ECC at different ages.

Figure 13 presents the influence of NLS content in the compressive strength of ECC at different ages. The early-age compressive strength (i.e., 7 days) was increased from 20 MPa to 24 MPa when the NLS content was increased from 0% to 2%. This result suggests that when the degree of hydration was low, the formation of ettringite due to the inclusion of NLS filled the pores in the cement matrix and thus led to a denser microstructure. However, for the 28-day compressive strength, the inclusion of NLS resulted in a lower strength of ECC, with a reduction of up to 30%. Arel and Aydin [73] indicated that the addition of NLS in cement leads to a reduced surface tension, which causes air entrainment and strength reduction. At 90 days, the strength development due to the pozzolanic reaction partially compensated the strength reduction due to air entrainment. The EECs with 0.5% of NLS achieved the lowest compressive strength at the 28-day and 90-day compared to other specimens. As discussed above, the LS consumption would be the highest at 0.5% NLS dosage among the considered mixtures. The semipermeable layer on the cement grains formed by the LS anions could slow down the migration of pore solution [66] and thus reduce the rate of cement hydration and the subsequent pozzolanic reaction. The slowed cement hydration could be again evident from the least development of the autogenous shrinkage of FA1.2-0.5% as shown in Figure 10. The decreased rate of cement hydration could be the cause of the reduced compressive strengths.

#### 3.4.2. Tensile Strength

**Figure 14:**
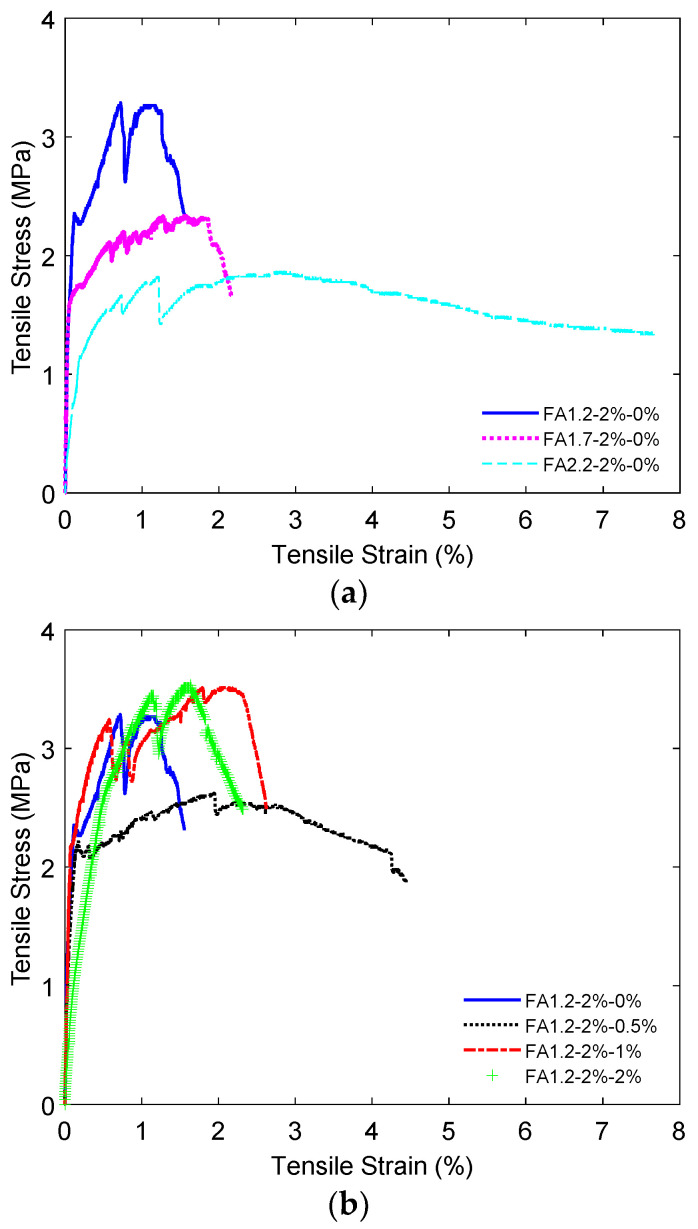
Tensile stress-strain relationships for ECC with *V_f_* = 2%: (**a**) Different FA contents; (**b**) Different NLS contents.

**Figure 15:**
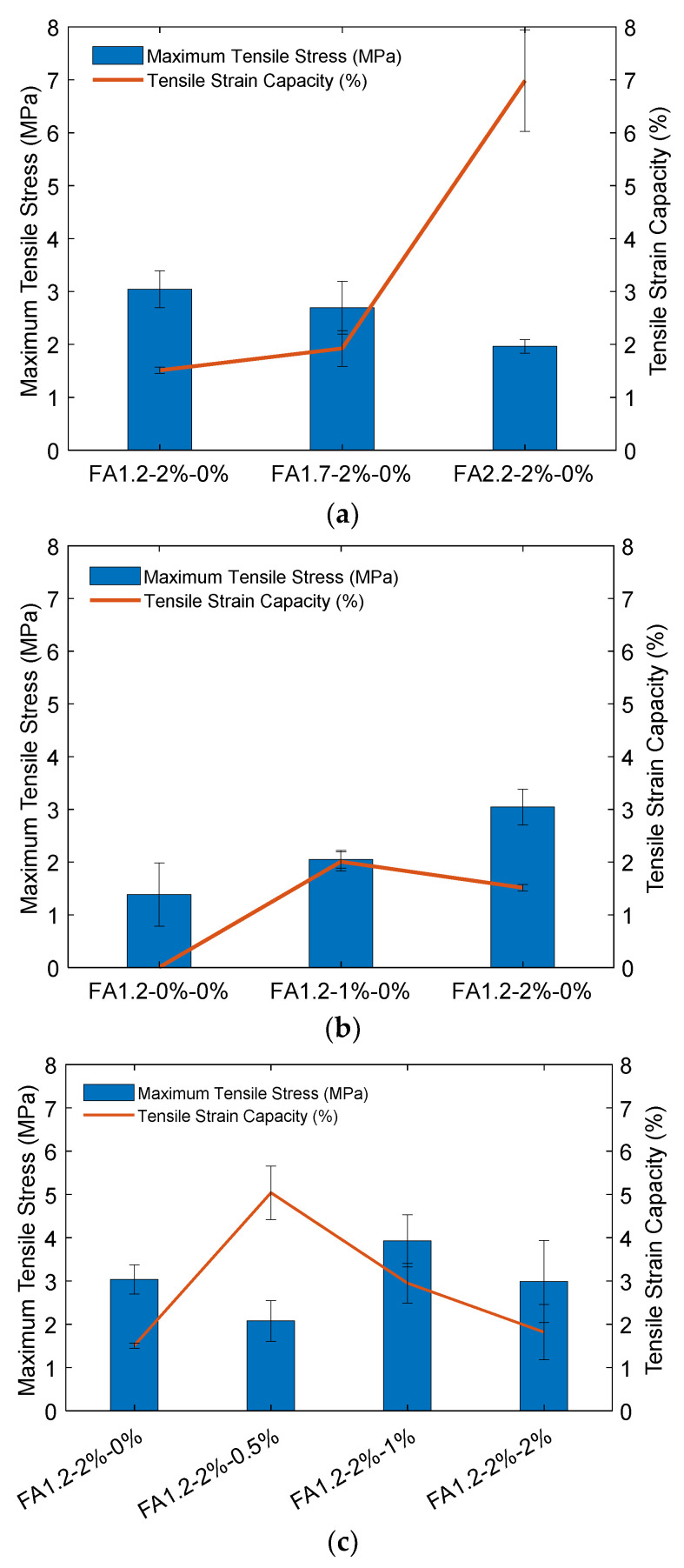
28-day tensile strengths and strain capacities of ECC specimens: (**a**) Influence of FA content; (**b**) Influence of fiber content; (**c**) Influence of NLS content.

Figure 14 shows the representative 28-day tensile stress-strain relationships for the ECC specimens with *V_f_* = 2%. It can be seen that ECC specimens exhibited tensile strain hardening behavior regardless of the FA and NLS contents. The 28-day tensile strength and strain capacities of ECC under the influences of FA/C ratio, fiber content, and NLS content are plotted in Figure 15. It can be seen in Figure 15a that although increasing the FA/C ratio from 1.2 to 2.2 reduced the tensile strength from 3.0 MPa to 2.0 MPa due to the lower cement content, it substantially enhanced the tensile strain capacity from 1.5% to 7.0% due to the reduced interfacial bond between fibers and the surrounding matrix [25]. Figure 15b shows that the tensile strength of ECC was effectively enhanced from 1.4 to 3.0 MPa when *V_f_* was increased from 0 to 2%. However, the higher tensile strength due to the increased *V_f_* from 1% to 2% slightly degraded the tensile strain capacity.

Figure 15c shows that increasing the NLS content from 0 to 2% did not cause significant influence in the tensile strength of ECC, likely because the tensile strength was mainly impacted by the bridging effect of fibers and the amount of C-S-H gels. As for the strain capacity, a small dosage of 0.5% NLS considerably enhanced the result from 1.5% to 5.0% This is likely due to the reduced strength and toughness of the cementitious matrix. As a result, the fracture energy required for crack initiation was reduced and thus promoting the multiple cracking behavior and the associated strain capacity. However, when the amount of NLS was further increased to 2%, the strain capacity of ECC degraded to 1.8%. This could be because the increased NLS content accelerated ettringite formation, which reduced the cohesiveness of the matrix and caused volumetric instability of the matrix [65].

## 4. Conclusions

The study showed that the initial and final setting times increased along with the NLS content of up to 1.0% and then remained relatively stable with a further higher NLS content to 2.0%. The development of HVFA-ECC’s drying shrinkage was governed by the first 14 days, and the autogenous shrinkage consistently developed for more than 28 days regardless of the fly ash content. Other important findings can be summarized as below.

(1) While the drying shrinkage of ECC was slightly increased with the higher FA/C ratio and fiber content, it was reduced by approximately 10% after the inclusion of NLS at 0.5%. However, the beneficial influence of NLS in reducing the drying shrinkage gradually decreased with the further higher NLS content.
(2) The inclusion of 0.5% NLS led to the least autogenous shrinkage and strengths, which could have resulted from the largest LS uptake by cement grains among the considered mixtures that slowed the cement hydration.
(3) Increasing the FA/C ratio from 1.2 to 2.2 reduced the 28-day comprehensive strength of ECC by about 60%. At 90 days, the low early-strength due to the use of a high FA/C ratio was partially compensated by the strength development due to the pozzolanic reaction. Moreover, when *V_f_* was increased from 0% to 2%, the 28-day compressive strength was considerably increased from 35 MPa to 62 MPa.
(4) Contrary to the negative impact on the strength, increasing the FA/C ratio from 1.2 to 2.2 substantially enhanced the tensile strain capacity of ECC by about five times. Furthermore, while the inclusion of NLS generally had minor influence in the tensile strength of ECC, it considerably enhanced the tensile strain capacity of ECC by more than two folds. Particularly, the inclusion of NLS at 0.5% led to the optimal enhancement in the tensile strain capacity, which was enhanced from 1.5% to 5.0%.
(5) The 7-day compressive strength of ECC was increased by about 20% after the inclusion of NLS. However, for the 28-day compressive strength, the inclusion of NLS resulted in a lower strength of ECC, with a reduction of up to 30%. At 90 days, the strength development due to the pozzolanic reaction helped compensate the strength reduction due to the formation of ettringite when NLS was present.

## Figures and Tables

**Table 1:** Mixture proportions of ECC by weight.

ECC Material	OPC	FA	SS	Fiber *	NLS
FA1.2-2%-0%	1	1.2	0.8	2	0
FA1.7-2%-0%		1.7			
FA2.2-2%-0%		2.2			
FA1.2-0%-0%		1.2		0	
FA1.2-1%-0%				1	
FA1.2-2%-0.5%				2	0.005
FA1.2-2%-1%					0.01
FA1.2-2%-2%					0.02

* The cement content is 382 kg/m^3^ for all mixtures, and PVA fiber is proportioned by volume *V_f_* %.

**Table 2:** Compositions (wt. %) of FA and NLS.

	CaO	SiO_2_	Al_2_O_3_	Fe_2_O_3_	P_2_O_5_	K_2_O	TiO_2_	MgO	Na_2_O	SO_3_	NH_4_^+^
FA	4.37	64.69	19.03	8.34	0.01	2.01	0.86	2.31	1.22	0.12	0.07
NLS	37.04	50.78	3.57	0.10	0.05	0.05	-	1.72	2.62	3.93	-

## Data Availability

The raw data required to reproduce these findings cannot be shared at this time due to time limitations. The processed data required to reproduce these findings cannot be shared at this time due to time limitations.
